# Phylogeography and the population genetic structure of flowering cherry *Cerasus serrulata* (Rosaceae) in subtropical and temperate China

**DOI:** 10.1002/ece3.6765

**Published:** 2020-09-13

**Authors:** Xian‐Gui Yi, Jie Chen, Hong Zhu, Yong‐Fu Li, Xue‐Xia Li, Meng Li, Yi‐Fan Duan, Lin Chen, Xian‐Rong Wang

**Affiliations:** ^1^ Co‐Innovation Center for the Sustainable Forestry in Southern China; Cerasus Research Center College of Biology and the Environment Nanjing Forestry University Nanjing Jiangsu China

**Keywords:** *Cerasus serrulata*, genetic diversity, genetic structure, phylogeography, resource utilization and conservation, taxonomic treatment

## Abstract

*Cerasus serrulata* (Rosaceae) is an important flowering cherry resource which is valuable for developing new cultivars of flowering cherries. It is broadly distributed and possesses abundant variations. In this study, phylogeographic analysis was conducted to reveal the evolutionary history to better understand the genetic diversity and genetic structure of *C. serrulata* so as to provide more accurate molecular insights into better conservation and utilization of the germplasm resources. A total of 327 individuals from 18 wild populations were collected. Three chloroplast DNA (cpDNA) fragments (matK, trnD‐E, and trnS‐G) and the nuclear internal transcribed spacer (ITS) were utilized. The results showed a high genetic diversity at both species level and population level of *C. serrulata*. High genetic differentiation and the existence of the phylogeographic structure were detected. No significant expansion events were discovered. Two geographic lineages were inferred. One was confined to the Qinling Mountains and the Taihang Mountains. The other was from the Wuling Mountains to the Jiangnan Hilly Regions and then went northeast to the coast of Asia. In addition, some taxonomic treatments of the *C. serrulata* complex are discussed and reconsidered. Conservation and utilization strategies of wild *C. serrulata* germplasm resources were recommended.

## Graphical abstract

*Cerasus serrulata* (Rosaceae) distributes in the main subtropical and temperate China as almost the most widely distributed cherry. It shows variable morphological traits, especially in ornamental traits, making it a significantly important cherry resource. We covered the major distributions and collected 18 populations to study the phylogeography of C. serrulata using three cpDNA fragments (matK, trnD‐E, trnS‐G) and the nuclear ITS to see its spatiotemporal evolutionary pattern to bring insights into evolutionary history of the genus and of plants within this region. We revealed the genetic structure of C. serrulata which also brought taxonomic proofs to the controversial treatments of the *C. serrulata* complex, and finally found two geographical lineages and inferred the evolutionary routines of *C. serrulata*.

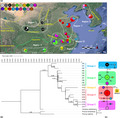

## 1 INTRODUCTION

Flowering cherry is an excellent ornamental flowering tree and is very popular around the world. Nowadays, most cultivars of flowering cherries are mainly developed from Japan. Based on our accumulated observation and study on the *Cerasus* resource in China and Japan, we believe *C. serrulata* to be one of the breeding origins of many of the good flowering cherry cultivars. Thus, making good use of this *C. serrulata* resource will contribute significantly to the development of new flowering cherry cultivars.

*C. serrulata* (Lindl.) G. Don ex London (Rosaceae) has a wide distribution range from west to east in China and is also distributed in the Korean Peninsula and Japan (Li & Bartholomew, 2003). It also covers a broad latitude range from about 24°N to 45°N (Li et al., 2014). Various environments are included in this broad area, including mountains, hilly regions, plains, islands, and inland and coastal areas of Asia. This distribution range is also mainly inside the subtropical and temperate area. Thus, such diverse habitats and different climates should have generated ample genetic resources in *C. serrulata*, which is of great value to the breeding of new flowering cherry cultivars (Yi et al., 2018). However, along with the high variations, there are many transition morphological characteristics that result in taxonomic controversies and which cause further trouble in records of the correct breeding procedure and making full use of the species resource. Although simple sequence repeats (SSRs) were used to study the genetic diversity and structure of *C. serrulata* (Chen, 2016; Yi et al., 2018), measurement at the base level, which is believed to provide deeper and better precision, is lacking.

A phylogeographic study is therefore a good choice for discovering more in‐depth and clearer information about the germplasm resources of *C. serrulata*. Phylogeography focuses on the gene genealogy spatiotemporal pattern of related species or intraspecies as well as how the pattern was formed (Bai & Zhang, 2014; Hickerson et al., 2010). Chloroplast DNA (cpDNA) sequences are generally used as it is uniparentally inherited (cpDNA in angiosperm is usually paternally inherited); this can provide a clearer evolutionary history without the effects of genetic recombination. Nuclear sequences (especially ITS) are also effective and can provide additional information. Thus, phylogeographic analysis using sequence data can provide information about the genetic diversity and structure at the base level, rather than a fragment length of traditional molecular markers, which ought to provide more abundant and accurate insights. In addition, divergence time and expansion time of subsets of a germplasm resource can be estimated based on the base substitution rate of nuclear sequences. Combining geological events at a corresponding time, the evolutionary history of geographic groups of a species can be inferred (Hickerson et al., 2010). Therefore, a more efficiently explained genetic structure and genetic diversity of a species can be revealed.

In phylogeographic analysis, glacial refugia play an important role, since they harbored species, allowing them to survive the harsh environment during the glacial period, and were the origin places of population colonization after glaciation; thus, they have eminent effects on shaping the contemporary distribution pattern of species and their genetic diversity and genetic structure (Chung, López‐Pujol, & Chung, 2017). *C. serrulata* is mainly distributed within subtropical and temperate China. The Nanling Mountains and the Qinling Mountains in subtropical China are believed to play dual roles, first as dispersal corridors in the east–west direction and second as glacial refugia during the late Quaternary (Guo, Wang, Bao, Bai, & Ge, 2014; Tian et al., 2018; Wulufu, 1964; Zhou, 1997). The main Korean mountain range (the Baekdudaegan (BDDG)) is also thought to be a glacial refugium, mainly for the boreal and temperate flora of northeastern Asia (Chung et al., 2017). In addition to those large mountains, microrefugia such as smaller massifs or lowland sites are also of great importance for sustaining species. It was hypothesized that multiple microrefugia were located in the northern part of the southern refuge (24°N to 33°N; Wang & Ge, 2006).

Phylogeographic studies can also provide new clues about taxonomy, as genetic relationships are analyzed using sequence data. There are taxonomic controversies regarding *C. serrulata* as mentioned above. *C. serrulata* is a complex consisting of three varieties recorded in *Flora of China* (Li & Bartholomew, 2003): *C. serrulata* var. *serrulata*, *C. serrulata* var. *lannesiana,* and *C. serrulata* var. *pubescens*. In addition, there are several taxonomic treatments that have remained controversial. Zang (2017) described a new species in Laoshan Mountain (Qingdao, Shandong Province, China), which resembles *C. serrulata* but differs in leaf shape, sepal serrature, petal shape, and fruit color. Yi (2007) discovered a group of special *Cerasus* individuals in Huanggangshan Mountain (Wuyishan, Fujian Province, China). These individuals are similar to *C. serrulata*, but are predominantly short, and the leaves are red and small. There are also controversial taxonomic relationships between *C. serrulata* and some other species, such as *C. xueluoensis* (Nan, Wang, Tang, & Yi, 2013).

In this study, a total of 18 wild populations distributed in subtropical and temperate China and the Korean peninsula were collected in order to conduct a phylogeographic analysis of *C. serrulata*. The sampling has covered the majority of *C. serrulata* distributions. For the first time, three paternally inherited cpDNA fragments and the biparentally inherited nuclear ITS sequences were used together for phylogeographic analysis of *C. serrulata* to reveal the spatiotemporal distribution history, so as to better understand its genetic diversity and genetic structure. Suggestions on conservation and utilization of the germplasm resources are recommended. In addition, in this study, individuals described as *C. laoshanensis* (Zang, 2017) in Laoshan Mountain (Qingdao, Shandong, China) were collected as population LaoS, and individuals recorded by Yi (2007) in the Huanggangshan Mountain (Wuyishan, Fujian, China) were sampled as population HGS to allow discussion of the taxonomic problems, in order to provide more proof for further clarifying their relationships with *C. serrulata*.

## 2 MATERIALS AND METHODS

### 2.1 Population sampling

**Figure 1:**
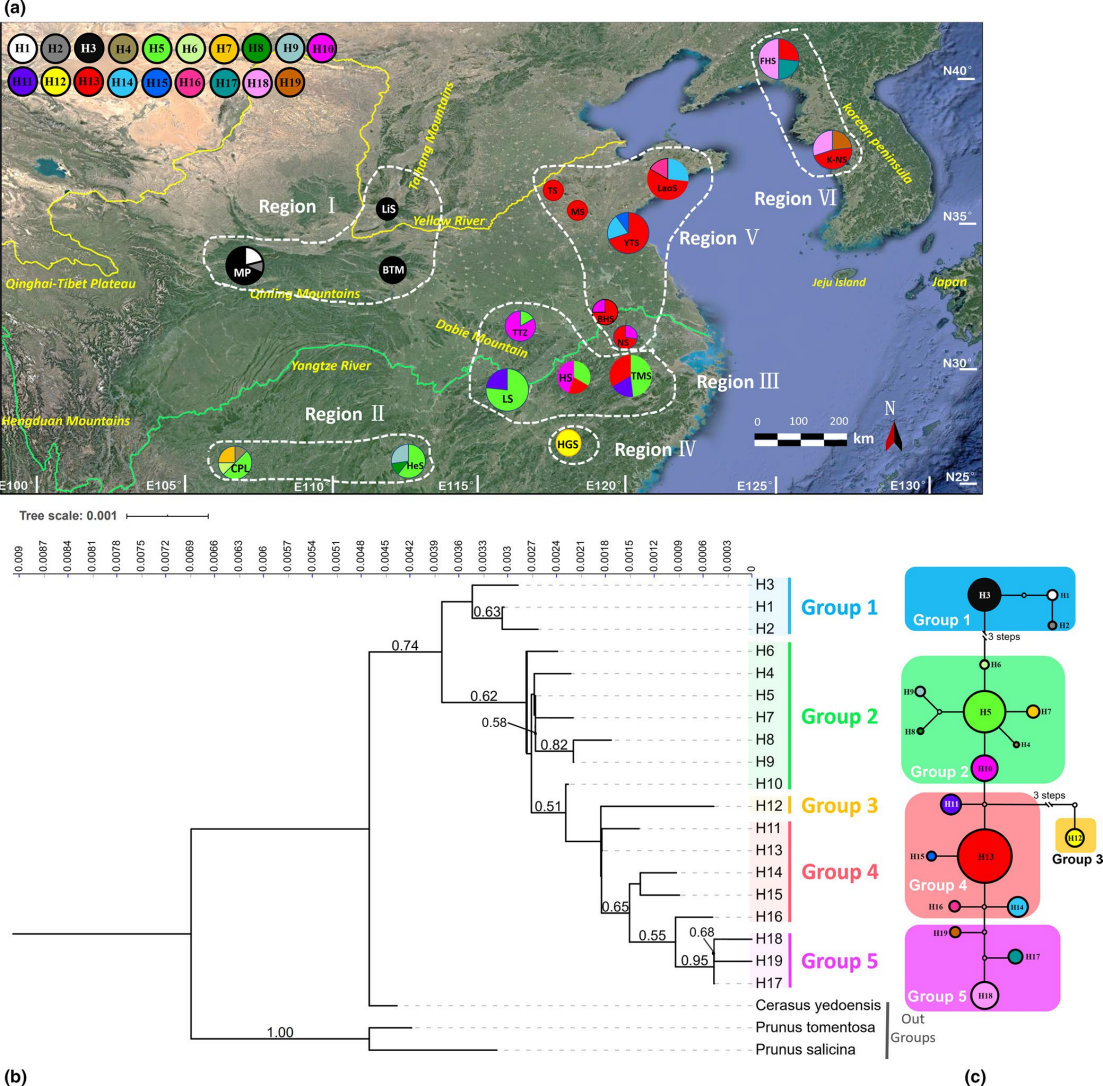
Haplotype structure of cpDNA sequences. (a) Geographic distribution of the cpDNA haplotypes in 18 natural populations of *C. serrulata*. Size of each circle represents the population size; color of the proportion in a circle indicates the type of haplotype, and the proportion corresponds to the number of individual(s) who have(s) the haplotype. (b) Neighbor‐joining tree of the cpDNA haplotypes of *C. serrulata*. Values of bootstraps larger than 0.5 are shown above the branches. Tree scale indicates branch length. (c) Network of cpDNA haplotypes. Each numbered circle (H1‐H19) represents a unique haplotype, and the size of the circle is proportional to the overall frequency of each haplotype in the entire sample of the species [Correction added on 27 September 2020 after first online publication: Figure 1 has been corrected in this version]

We conducted field investigations of *C. serrulata* according to specimen and literature records since 2010. A total of 327 individuals were collected from 18 wild populations, 17 in China and one in Korea (Table 1, Figure 1a). Our collections roughly covered the distributions of *C. serrulata*. Green, young, and healthy leaves were sampled from individuals at least 30 m apart and were quickly stored in silica gel. All samples were used for cpDNA analysis, while 174 were used for ITS analysis with a maximum accession number of 10 for each population (Table 1).

**Table 1:** Location, haplotypes, and genetic diversity of populations of *C. serrulata*

Symbol	Location	Geographic coordinate	Altitude (m)	Population size cpDNA/ITS	Haplotype diversity (*H*_d_) cpDNA/ITS	Nucleotide diversity *P*_i_ (×10^–3^) cpDNA/ITS	Haplotypes (No. of individuals)	Ribotypes (No. of individuals)
MP	Miaoping, Hanzhong, Shaanxi Province, China	106.680°E, 33.382° N	1,331	19/10	0.503/0.533	0.520/0.810	H1(4), H2(2), H3(13)	R6(4), R7(6)
LiS	Li Mt., Yuanqu, Shanxi Province, China	111.999°E, 35.340° N	753	9/9	0.000/0.000	0.000/0.000	H3(9)	R7(9)
BTM	Baotianman, Nanyang, Shanxi Province, China	111.930°E, 33.374° N	465	13/10	0.000/0.000	0.000/0.000	H3(13)	R7(10)
CPL	Changpoling, Guiyang, Guizhou Province, China	106.671°E, 27.658° N	1,357	16/10	0.700/0.733	0.400/2.820	H4(2), H5(8), H6(2), H7(4)	R1(3), R2(4), R4(3)
HeS	Heng Mt., Hengyang, Hunan Province, China	112.694°E, 27.291° N	1,024	15/10	0.533/0.556	0.340/2.520	H5(10), H8(2), H9(3)	R5(5), R4(5)
TTZ	Tiantangzhai, Jinzhai, Anhui Province, China	115.761°E, 31.164° N	785	12/10	0.303/0.000	0.140/0.000	H5(2), H10(10)	R4(10)
LS	Lu Mt., Jiujiang, Jiangxi Province, China	116.982°E, 29.545° N	1,109	30/10	0.370/0.533	0.510/2.420	H5(23), H11(7)	R4(6), R5(4)
HGS	Huanggangshan, Wuyishan, Fujian Province, China	117.767°E, 27.841° N	1802	10/10	0.000/0.000	0.000/0.000	H12(10)	R3(10)
HS	Huang Mt., Huangshan, Anhui Province, China	118.168°E, 30.130° N	1,124	18/10	0.680/0.356	0.460/1.610	H5(6), H10(4), H13(8)	R4(8), R5(2)
TMS	Tianmu Mt., Lin'an, Zhejiang Province, China	119.430°E, 30.343° N	1,185	27/10	0.647/0.467	0.630/2.120	H5(13), H11(5), H13(9)	R4(7), R5(3)
NS	Nanshan, Liyang, Jiangsu Province, China	119.514°E, 31.175° N	362	11/10	0.436/0.000	0.200/0.000	H10(3), H13(8)	R4(10)
BHS	Baohua Mt., Zhenjiang, Jiangsu Province, China	119.078°E, 32.138° N	165	12/10	0.409/0.000	0.190/0.000	H10(3), H13(9)	R4(10)
YTS	Yuntaishan., Lianyungang, Jiangsu Province, China	119.443°E, 34.719° N	410	30/10	0.476/0.000	0.440/0.000	H13(21), H14(6), H15(3)	R4(10)
TS	Tai Mt., Tai'an, Shandong Province, China	117.111°E, 36.232° N	627	8/8	0.000/0.000	0.000/0.000	H13(8)	R4(8)
MS	Mengshan Mt., Linyi, Shandong Province, China	117.958°E, 35.551° N	528	7/7	0.000/0.000	0.000/0.000	H13(7)	R4(7)
LaoS	Laoshan Mt., Qingdao, Shandong Province, China	120.602°E, 36.196° N	630	30/10	0.600/0.000	0.690/0.000	H13(17), H14(8), H16(5)	R4(10)
FHS	Phoenix Mt., Dandong, Liaoning Province, China	124.092°E, 40.429° N	448	30/10	0.646/0.000	0.800/0.000	H13(8), H17(7), H18(15)	R8(10)
K‐NS	Nanshan, Seoul, Korea	126.987°E, 37.548° N	205	30/10	0.660/0.356	1.090/0.540	H13(14), H18(9), H19(7)	R8(2), R9(8)
All				327/174	0.829/0.673	1.500/5.210	H1(4), H2(2), H3(35), H4(2), H5(62), H6(2), H7(4), H8(2), H9(3), H10(20), H11(12), H12(10), H13(109), H14(14), H15(3), H16(5), H17(7), H18(24), H19(7)	R1(3), R2(4), R3(10), R4(94), R5(14), R6(4), R7(25), R8(12), R9(8)

### 2.2 DNA extraction, PCR amplification, and sequencing

The genomic DNA was extracted by the CTAB‐SiO_2_ method (Nan, 2012; Zhang, Zhang, Zhu, Wang, & Huang, 2004). Three cpDNA fragments selected after screening, matK, trnD‐E, trnS‐G, and the nuclear ITS sequence, were amplified. Polymerase chain reaction (PCR) amplifications were carried out in a system of reaction mixtures of 25 μl, containing 12.5 μl of 2 × Taq PCR Master Mix (Tiangen, Beijing, China), 1 μl of each primer, 2 μl of DNA template, and 8.5 μl of ddH_2_O. The PCR procedure began at an initial denaturation of 5 min at 95°C, followed by 35 cycles of 45‐s denaturation at 94°C, 45‐s annealing at 56°C, 45‐s extension at 72°C, and ended at an extension of 8 min at 72°C. The PCR products were checked by a 1.0% agarose gel and were sent for sequencing on ABI Prism 3,730 Genetic Analyzer (Sangon, Shanghai, China).

### 2.3 Genetic diversity and phylogeographic analysis

All of the sequences were checked visually and edited manually using BioEdit version 7.09 (Hall, 1999), and then aligned using ClustalW in MEGAX (Kumar, Stecher, Li, Knyaz, & Tamura, 2018). The three cpDNA sequences were concatenated for subsequent analysis. Identification of cpDNA haplotypes (H) and ITS ribotypes (R), and calculation of their diversities (*H*_d_/*R*_d_) as well as nucleotide diversity (*P*_i_) were performed using DnaSP v6.0.01 (Rozas et al., 2017). Analysis of molecular variance (AMOVA) was conducted to estimate the genetic variances within and between populations and regions using Arlequin version 3.5 (Excoffier & Lischer, 2010). Genetic differentiation between regions was estimated using BAPS 6.0 (Cheng, Connor, Sirén, Aanensen, & Coraner, 2013). The network of cpDNA haplotypes and ITS ribotypes was drawn using TCS1.2.1 (Clement, Posada, & Crandall, 2000). Phylogenetic analysis was conducted using MEGAX (Kumar et al., 2018) to draw the neighbor‐joining tree with 1,000 bootstrap replications. The trees were edited using an online tool, Interactive Tree Of Life (iTOL v5) (Letunic & Bork, 2019). *Prunus salicina*, *C. tomentosa*, and *C. yedoensis* were taken as outgroups. All the above analysis involved both cpDNA and ITS sequences.

*G*_ST_, representing haplotype frequency, and *N*_ST_, representing haplotype difference, were calculated to examine the existence of population structure by PERMUT v. 2.0 with 10,000 permutations (Pons & Petit, 1996). *N*_ST_ > *G*_ST_, *p* < .05 indicates the presence of a phylogeographic structure. Genetic differentiation coefficient (*GammaSt*) between regions clustered using BAPS 6.0 was calculated using DnaSP v6.0.01 (Rozas et al., 2017). Mismatch distribution analyses of each group and all were conducted using analysis of population size changes in DnaSP v6.0.01 to test whether they were constant in size or experienced growth or decline (Rozas et al., 2017). Neutrality tests of Tajima's *D* and Fu's *F*_S_ were conducted to test population expansion using Arlequin ver. 3.5 with the number of simulated samples set to be 10,000 (Excoffier & Lischer, 2010). These analyses were only conducted with cpDNA sequences.

### 2.4 Divergence time dating

CpDNA haplotypes were used for analyzing the divergence time dating. PartitionFinder 2 (Lanfear, Frandsen, Wright, Senfeld, & Calcott, 2017) was utilized using greedy algorithm (Lanfear, Calcott, Ho, & Guindon, 2012) with PhyML (Guindon et al., 2010) to find the best partitioning scheme for the sequences and the best‐fit models for each partitioned subset. The branch lengths were set to be linked. Models were selected from all available substitution models in BEAST V1.10.4 (Suchard et al., 2018). Model selection was set to AICC. The divergence times were estimated by BEAST v1.10.4 (Suchard et al., 2018). Lacking fossil records, secondary calibration points according to the diversification of Rosaceae were applied to calibrate node ages. Divergence time dating was calculated in two steps with two trees, respectively. Five secondary calibration points were applied to the first tree: Rosaceae Crown, 95.09 Mya (Li et al., 2017; Zhang et al., 2017); Amygdaleae, 84.84 Mya (Zhang et al., 2017); Spiraeeae, 84.30 Mya (Zhang et al., 2017); Roseae + Rubeae, 59.51 Mya (Zhang et al., 2017); and Maleae Crown, 50.06 Mya (Li et al., 2017; Zhang et al., 2017) to evaluate the divergence time of narrow *Prunus* (node 1) and *Cerasus* (node 2). These two nodes were subsequently used to calibrate the node ages in the second tree of haplotypes of *C. serrulata* (Figure 3). According to the results of analysis using PartitionFinder 2, four subsets were set and their corresponding substitution model was GTR + I, HKY + I, GTR + G, and GTR + G, respectively, for the first tree, while HKY + I+G, HKY, JC and JC were selected for the four subsets of the second tree. BEAST was run several times to find the most suitable settings by checking the convergence in Tracer v1.7.1 (Rambaut, Drummond, Xie, Baele, & Suchard, 2018). Finally, the uncorrelated log‐normal relaxed clock and Yule process as for tree prior were selected, and length of chain in MCMC was set to 5 × 10^7^ for the first tree; the uncorrelated log‐normal relaxed clock and GMRF Bayesian Skyride of tree prior were selected with MCMC set to 5 × 10^7^ for the second tree. Maximum clade credibility (MCC) trees were constructed to create consensus trees summarizing the posterior tree distribution using the results of BEAST analyses by TreeAnnotator v1.10.4. Final phylogenetic trees were edited in FigTree v. 1.4.4.

## 3 RESULTS

### 3.1 Genetic diversity

The total length of aligned sequences for the three chloroplast fragments (matK, trnD‐E, and trnS‐G) was 2,161 bp with 26 variable sites, including 5 sites with alignment gaps, 8 singleton variable sites, and 13 parsimony informative sites in 327 individuals. The total length of ITS was 661 bp with 10 variable sites in 174 individuals. Results of the diversity analyses are shown in Table 1. At the species level, the haplotype diversity (*H*_d_) was 0.829 and the ribotype diversity (*R*_d_) was 0.673; the nucleotide diversity (*P*_i_) of cpDNA and ITS was 1.500 and 5.210, respectively. All the data suggested a high genetic diversity of *C. serrulata*. At population level, *H*_d_ varied from 0.000 to 0.700, while *R*_d_ varied from 0.000 to 0.733; *P*_i_ × 10^–3^ of cpDNA varied from 0.000 to 1.090, while that of ITS varied from 0.000 to 2.820. Population CPL showed the highest haplotype diversity both in cpDNA and in ITS regions.

At the region level, *H*_d_ varied from 0.000 to 0.690, and *R*_d_ varied from 0.000 to 0.733, while *P*_i_ varied from 0.000 to 0.980 for cpDNA and from 0.000 to 2.820 for ITS (Table 2). However, the regional grouping of populations by the two kinds of DNA fragments was a little different. The cpDNA analysis showed that Region VI possessed the highest genetic diversity, while Region IV had no diversity as it only contained one population, population HGS, and the population only had one specific haplotype. As for the ITS‐defined regions, the highest genetic diversity was in Region II with only one population CPL, which showed the highest haplotype and ribotype diversity both in cpDNA and in ITS sequences. However, there was no diversity in Region VI and Region V. Region VI was similar to that of cpDNA, and only contained one unique population, HGS, while Region V included the wide populations in north China, and the genetic diversity was very different from that of cpDNA.

**Table 2:** Population and haplotype distribution and genetic diversity of the six regions

Region	DNA type	Population distribution	Group size	Haplotype diversity *H*_d_/*R*_d_	Nucleotide diversity *P*_i_ (×10^–3^)	Haplotypes/Ribotypes (No. of individuals)
Region I	cpDNA	MP, LiS, BTM	41	0.266	0.280	H1(4), H2(2), H3(35)
	ITS	MP, LiS, BTM	29	0.246	0.370	R6(4), R7(25)
Region II	cpDNA	CPL, **HeS**	31	0.645	0.410	H4(2), H5(18), H6(2), H7(4), H8(2), H9(3)
	ITS	CPL	10	0.733	2.820	R1(3), R2(4), R4(3)
Region III	cpDNA	**TTZ**, LS, HS, TMS	89	0.669	0.550	H5(44), H10(14), H11(12), H13(17)
	ITS	**HeS**, LS, HS, TMS	40	0.467	2.120	R4(26), R5(14)
Region IV	cpDNA	HGS	10	0.000	0.000	H12(10)
	ITS	HGS	10	0.000	0.000	R3(10)
Region V	cpDNA	NS, BHS, YTS, TS, MS, LaoS	98	0.467	0.450	H10(6), H13(70), H14(14), H15(3), H16(5)
	ITS	**TTZ**, NS, BHS, YTS, TS, MS, LaoS	65	0.000	0.000	R4(65)
Region VI	cpDNA	FHS, K‐NS	60	0.690	0.980	H13(22), H17(7), H18(24), H19(7)
	ITS	FHS, K‐NS	20	0.505	0.7560	R8(12), R9(8)

Note

Population symbols in bold indicate the differences in grouping of both DNA types of fragments.

### 3.2 Genetic structure

Analysis of molecular variance (AMOVA) revealed a high differentiation level of both cpDNA (*F*_st_ = 0.72461) and ITS (*F*_st_ = 0.88730) in *C. serrulata* (Table 3). The most variation was among regions, indicated by the percentage of 67.78% cpDNA variation and 87.6% ITS variation. Variation among populations within regions was very small (cpDNA: 4.68%; ITS: 1.13%). AMOVA indicated a genetic structure in the *C. serrulata* population. The phylogeographic structure was also affirmed by comparing the haplotype frequency and haplotype difference (*N*_ST_ = 0.434 > *G*_ST_ = 0.311, *p* < .05).

**Table 3:** AMOVA based on cpDNA and ITS data for *C. serrulata*

Source of variation	*df*	Sum of squares	Variance components	Percentage of variation
	cpDNA/ITS	cpDNA/ITS	cpDNA/ITS	cpDNA/ITS
Among regions	5/5	425.126/254.155	1.61247/1.90351 Va	67.78/87.60
Among populations within regions	12 /12	31.276/5.748	0.11136/0.02446 Vb	4.68/1.13
Within populations	309 /156	202.441/38.200	0.65515/0.24487 Vc	27.54/11.27
Total	326 /173	658.844/298.103	2.37899/2.17284	100/100
Fixation Indices	*F*_SC_	0.14529/0.09081		
	*F*_ST_	0.72461/0.88730		
	*F*_CT_	0.6778/0.87605		

Note

Distance method: Pairwise difference.

**Figure 2:**
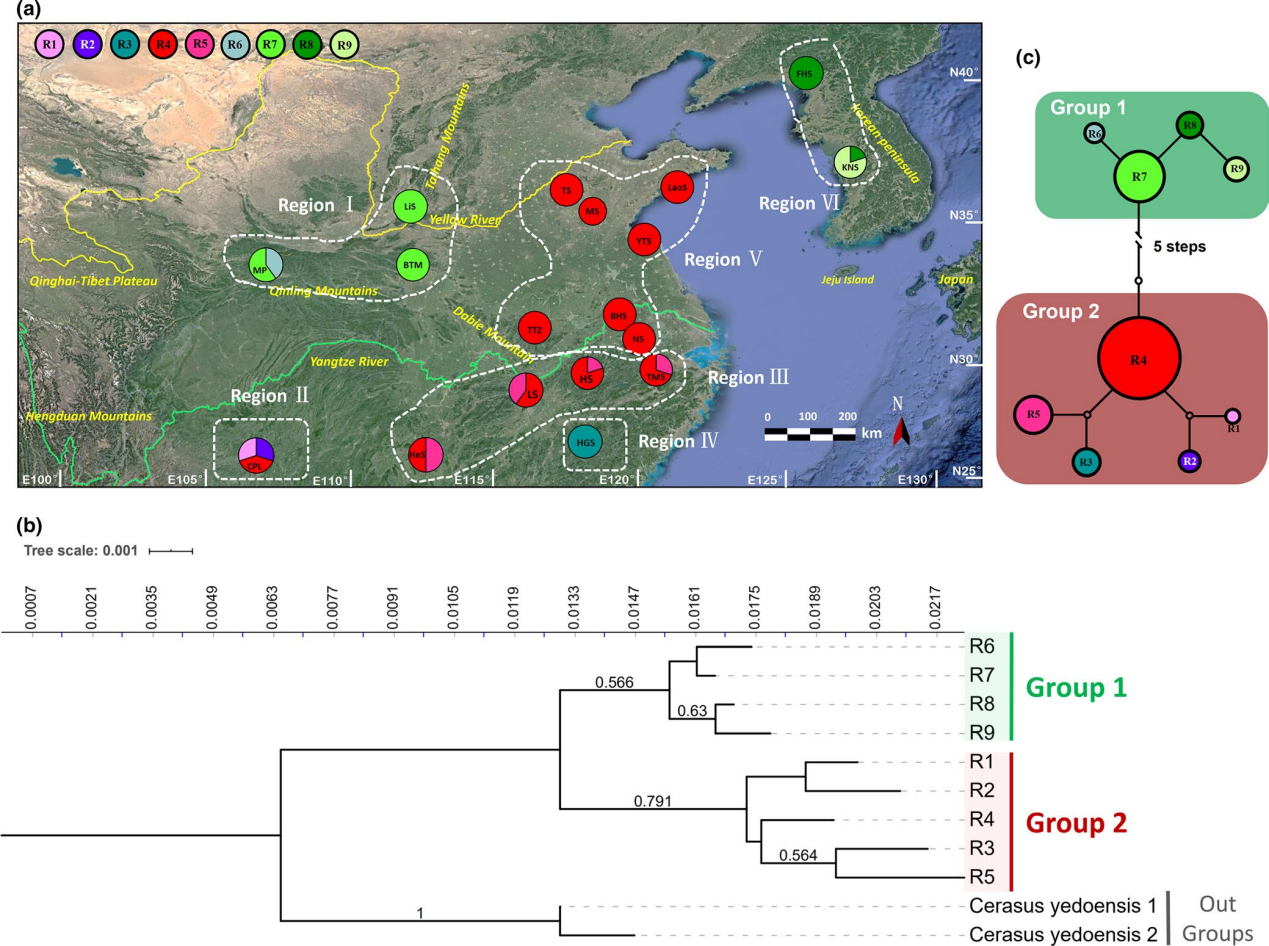
Ribotype structure of ITS sequences. (a) Geographic distribution of the ribotypes based on ITS sequences in 18 natural populations of *C. serrulata*. Size of each circle represents the population size; color of the proportion in a circle indicates the type of haplotype, and the proportion corresponds to the number of individual(s) who have(s) the haplotype. (b) Neighbor‐joining tree of the ribotypes based on ITS sequences. Values of bootstraps larger than 0.5 are shown above the branches. Tree scale indicates branch length. (c) Network of the ribotypes based on ITS sequences. Each numbered circle (R1‐R9) represents a unique ribotype, and the size of the circle is proportional to the overall frequency of each ribotype in the entire sample of the species

Analysis using BAPS identified six groups of regions of the 18 populations of *C. serrulata* of both DNA fragments, but there were some differences in the groupings (Table 2, Figures 1a, 2a). As for cpDNA sequences, Region I contained population MP, LiS, and BTM in the northwest region. Region II consisted of population CPL and HeS in the southwest region. Region III was distributed in the Jiangnan Hilly Region with population TTZ, LS, HS, and MS. Region IV located within the Wuyi Mountains and had only one unique population HGS who had only one haplotype or ribotype and shared none with other regions. Region V comprised populations on the plain on the east coast of Asia (NS, BHS, YTS, TS, MS, and LaoS) and is the largest group. Region VI was composed of populations FHS and K‐NS, ranged from the Korean Peninsula to almost the junction between China and North Korea. As for ITS sequences, population HeS (grouped in the southwest region, Region II by cpDNA analysis) was set to Region III, along with the Jiangnan Hilly Region; population TTZ was clustered into Region V on the plain on the east coast of Asia, rather than to be set to Region III in the Jiangnan Hilly Region. This seems to indicate a geographic lineage from the southwest region to the east coast of Asia, and the Jiangnan Hilly Region is the transition area; thus, populations at junctions of its two sides (HeS and TTZ) showed an ambiguous station in grouping.

In both sequence analyses, population LaoS, representing *C. laoshanensis* (Zang, 2017), showed no dominant distinctiveness, though it owned a unique haplotype (H16) that no other populations had. Population HGS appeared to have remarkable specificity since it possessed a special haplotype (H12) in the cpDNA sequences and a unique ribotype (R3) in the ITS sequences, and made up a region (Region IV) itself from other populations.

### 3.3 Genetic differentiation and gene flow among regions

Pairwise genetic differentiation coefficients (*GammaSt*) between different regions were estimated, and gene flow was calculated according to the equation *N*m = (1/*GammaSt*−1)/2 (Zhu et al., 2019) to show genetic differentiation conditions between these regions (Tables 4, 5). Analysis of ITS sequences showed a relatively higher differentiation and lower gene flow than cpDNA. This might be because of a smaller sampling size of ITS sequences. Both cpDNA and ITS sequences identified a highest gene flow and lowest differentiation between Region II and Region III (*N*m_cpDNA_: 3.29564; *N*m_ITS_: 1.69356), followed by Region III and Region V (*N*m_cpDNA_: 1.48799; *N*m_ITS_: 1.50,000). Region I showed a dominantly isolated condition from others with high differentiation coefficients and low gene flow revealed by both DNA fragments. Region IV and Region V appeared extremely differentiated from each other by ITS as no gene flow was observed between them. Region VI appeared to have much less genetic exchanges with Region V through ITS gene flow than chloroplast gene flow.

**Table 4:** Pairwise values of genetic differentiation coefficient (*GammaSt*, above diagonal) and gene flow (*N*m, below diagonal) of cpDNA sequences among six regions of *C. serrulata*

	Region I	Region II	Region III	Region IV	Region V	Region VI
Region I		0.71923	0.64401	0.85279	0.73592	0.69413
Region II	0.19519		0.13173	0.74200	0.43986	0.48106
Region III	0.27639	3.29564		0.39345	0.25151	0.39542
Region IV	0.08631	0.17385	0.77081		0.37250	0.37554
Region V	0.17942	0.63673	1.48799	0.84228		0.28061
Region VI	0.22033	0.53937	0.76448	0.83142	1.28183	

Note

Gene flow *N*m was estimated through *N*m = (1/*GammaSt* − 1)/2.

**Table 5:** Pairwise values of genetic differentiation coefficient (*GammaSt*, above diagonal) and gene flow (*N*m, below diagonal) of ITS sequences among six regions of *C. serrulata*

	Region I	Region II	Region III	Region IV	Region V	Region VI
Region I		0.55397	0.47974	0.86685	0.92152	0.62852
Region II	0.40258		0.22794	0.56250	0.54480	0.36943
Region III	0.54223	1.69356		0.36571	0.25000	0.48940
Region IV	0.07680	0.38889	0.86720		1.00000	0.85246
Region V	0.04258	0.41777	1.50000	0.00000		0.88262
Region VI	0.29552	0.85344	0.52166	0.08654	0.06650	

Note

Gene flow *N*m was estimated through *N*m = (1/*GammaSt*−1)/2.

### 3.4 Distribution pattern and phylogenetic relationship of cpDNA haplotypes

A total of nineteen haplotypes were detected (Figure 1a, Tables 1, 2), of which eight were unique to only one population. Haplotype H13 was the most common, followed by H5. Number of haplotypes in each population ranged from one to four, but five populations owned only one kind of haplotype.

Combining the geographic distribution pattern (Figure 1a), NJ tree (Figure 1b), and the network (Figure 1c) of the 19 haplotypes, and also referring to BAPS analysis of the population structure, five groups from the haplotypes of cpDNA sequences were clustered. Group 1 consisted of H1, H2, and H3 and was relatively isolated and distinct from the northwest region (the Qinling Mountains and the Taihang Mountains) with population MP, LiS, and BTM. Group 2 contained seven haplotypes (H4‐H10) with H5 being the most dominant. H5 distributed from the southwest region (Region II, around the Wuling Mountains) to the Jiangnan Hilly Region (Region III) and connected the two regions. Group 3 is a mono‐haplotype group with a unique haplotype H12 located in the Wuyi Mountains (Region IV). The haplotype range of Group 4 (H11, H13‐H16) was the furthest. This group was distributed from the Jiangnan Hilly Region (Region III), northward to the plain on the east coast of Asia (Region IV), and continued eastward until the Korean Peninsula (Region VI). These regions were mainly linked by the most common haplotype of Group 4: H13. Group 5 contained three haplotypes: H17‐H19. It is distributed along the east coast of the Bohai Sea, including the junction of China and North Korea, and the Korean Peninsula (Region VI). It can be seen that H5 and H13 mainly link up the great area from the southwest to the northeast, containing regions from II to VI, covering the Wuling Mountains, the Jiangnan Hilly Region, the plain on the east coast of Asia, and junction of China and North Korea, as well as the Korean Peninsula together.

### 3.5 Distribution pattern and phylogenetic relationship of ITS ribotypes

A total of nine ITS ribotypes were identified in the 174 individuals (Figure 2a, Tables 1, 2). Of all the ribotypes, five were unique only to one population, and ribotype R4 was the most standard ribotype which covered 12 populations, and was the unique ribotype for seven populations.

The distribution pattern (Figure 2a), the phylogenetic tree (Figure 2b), and the network (Figure 2c) together exhibited a structure simply with two groups of these ribotypes. Group 1 incorporated four ribotypes (R6‐R9) with R7 being the main ribotype. Ribotype distribution of Group 1 was interrupted by the North China Plain and the Bohai Sea into two main regions: the northwest region in the Qinling Mountains and the Taihang Mountains (Region I), and the junction of China and North Korea as well as the Korean Peninsula (Region VI). Group 2 consisted of five ribotypes (R1‐R5) with R4 being the most extraordinary ribotype. This group involved 13 populations (Region II to Region V), ranging far from the southwest region in Wuling Mountains, through the Jiangnan Hilly Region and the Wuyi Mountains, to the plain on the east coast of Asia.

### 3.6 Divergence time dating

**Figure 3:**
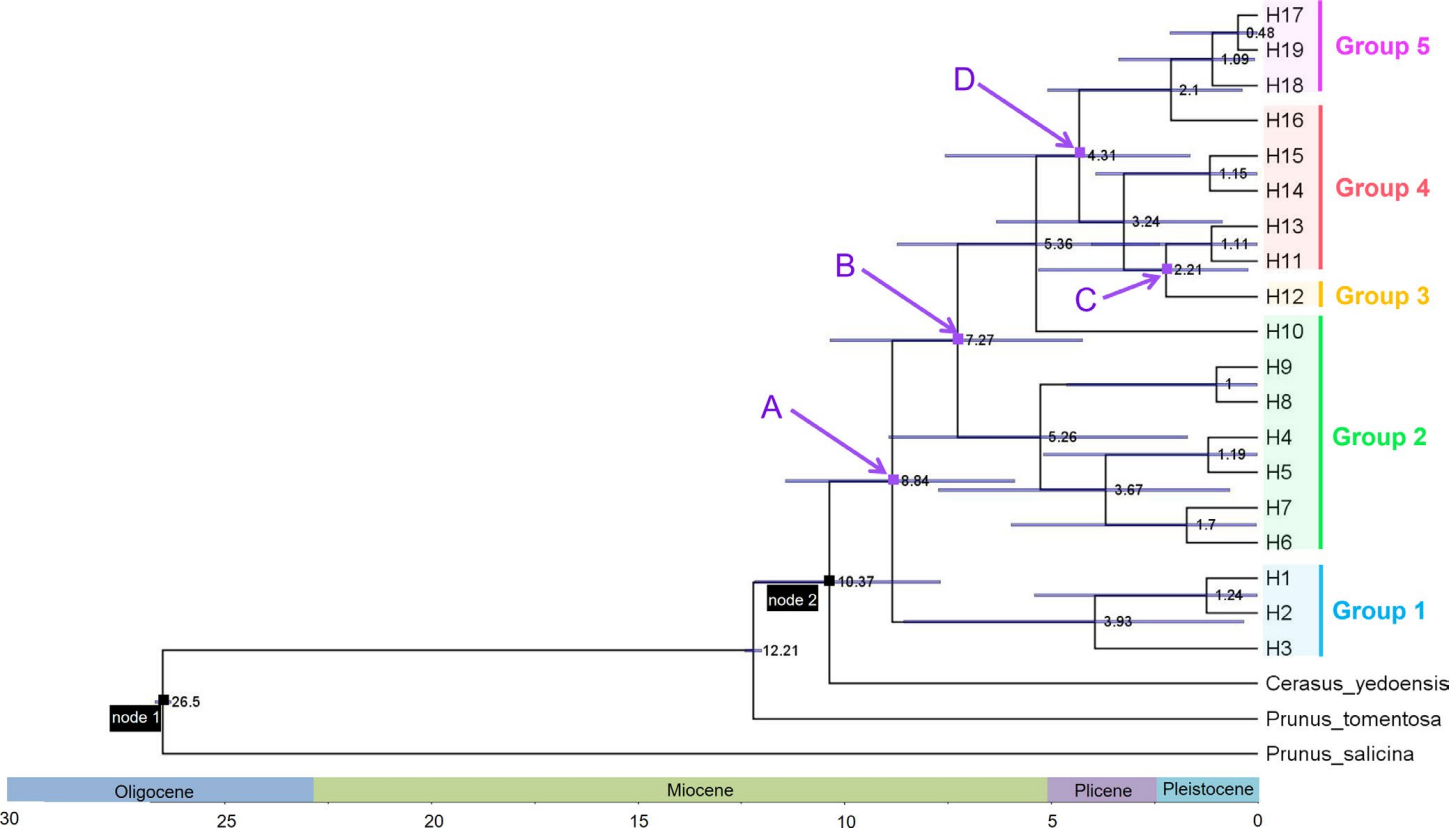
BEAST‐derived chronogram of *C. serrulata* based on cpDNA sequences. Numbers at each node indicate the node age (million years ago, mya). Ages of node 1 and node 2 have been calibrated. The blue bars illustrate the extent of the 95% highest posterior density (HPD) credibility intervals for each divergence time. Ages of nodes marked in purple spots and pointed by purple arrows marked from A to D correspond to the divergence time of Group 1 to Group 5, respectively

The divergence time of the most recent common ancestor (TMRCA) of *C. serrulata* was estimated to be around 10.37 mya (95% HPD: 7.67–12.17) (Figure 3), near the middle of the Miocene. The crown time of *C. serrulata* was around 8.84 mya (node A). Group 1, concerning Region I in the Qinling Mountains and Taihang Mountains, began its divergence from other groups around 8.84 mya. Group 2 began to diverge out around 7.27 mya (node B). Group 3 (Region IV, population HGS in the Wuyi Mountains) started to separate at approximate 2.21 mya (node C). Group 4 and Group 5 (Region VI, mainly in the Korean Peninsula) started to separate away from each other around 4.31 mya (node D).

### 3.7 Population dynamics

Mismatch distribution is the distribution of the observed pairwise nucleotide site differences. Mismatch distribution analysis in BAPS shows the observed mismatch distribution and the expected values in growing and declining populations (Rogers & Harpending, 1992, equation 4). Under the assumption of neutrality, a significantly negative value for Tajima's *D* and Fu's *F*_S_, along with a unimodal mismatch distribution curve, indicates a population expansion (Fu & Li, 1993; Tajima, 1989, 1996).

**Figure 4:**
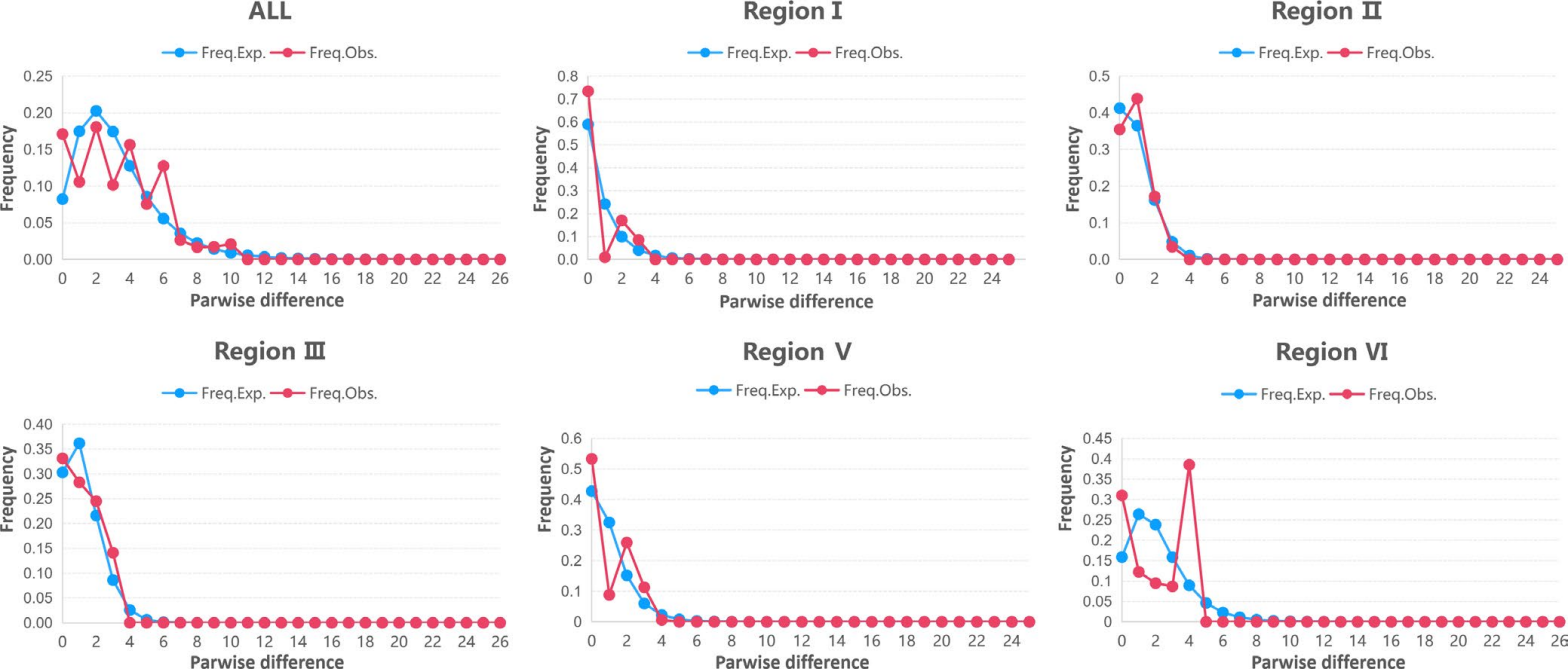
Mismatch distribution curve of the six regions and all populations of *C. serrulata*. The blue line shows expected values (Exp.), the red line represents the observed values (Obs.) under a model of sudden expansion/spatial expansion. Mismatch distribution curve of Region IV was missed because of no polymorphisms in the data as the region only possessed only one haplotype

In the neutrality test, the value of Tajima's *D* and Fu's *F*_S_ for all and the six *C. serrulata* groups was mainly positive or unremarkable negative (Region II), but *p* > .05 (Table 6), denoting that the hypothesis of neutral evolution was not to be excluded. The mismatch distribution curve for all was multimodal which rejected the expansion assumption (Figure 4). Of all the six regions, only Region II showed a weak unimodal curve, while others did not; thus, no obvious evidence for population expansion was determined.

**Table 6:** Neutrality test for the six regions of *C. serrulata*

Statistics	Region I	Region II	Region III	Region IV	Region V	Region VI	All
Tajima's *D*	−0.290	−0.790	1.856	0.000	0.003	2.271	0.05787
p‐value	0.421	0.241	0.972	1.000	0.581	0.993	0.5505
Fu’S *F*_S_	0.670	−0.726	2.835	0.000	0.726	5.497	−0.12841
p‐value	0.621	0.320	0.899	NA	0.684	0.966	0.555

Note

NA: The value is not available because there is only one haplotype in the region.

## 4 DISCUSSION

### 4.1 Genetic diversity

*Cerasus serrulata*, of the genus *Cerasus*, is a widely distributed species in China. It is also distributed in the subtropical and temperate monsoon climate zones. With such a broad distribution, it is expected to have a higher genetic diversity than other *Cerasus* species. Both cpDNA (*H*_d_ = 0.829, *P*_i_ = 1.500) and ITS (*R*_d_ = 0.673, *P*_i_ = 5.210) analysis proved a high genetic diversity of *C. serrulata*. This is higher than wild *C. pseudocerasus* (cpDNA: *h* = 0.557, *π* = 2.09), which is also a widely distributed *Cerasus* species (Chen et al., 2015). The diversity based on ITS sequences was lower than *C. dielsiana* (ITS: *H*_d_ = 0.879, π = 3.56), which is relatively widely distributed (Zhu et al., 2019). This may be a result of less sampling, rather than a real reflection of a lower diversity level. The broad spreading pattern must be a significant reason for the high diversity since diverse topographies within the distribution pattern of *C. serrulata* such as alps, mountains, and hills, as well as plains, have provided varying habitats for *C. serrulata*. In addition, the different climate zones have further increased the variances. All these multifarious environments must have impelled the variations and high diversity during adaptation in the diffusion of *C. serrulata*. The large distribution also indicates that *C. serrulata* has a strong ability to adapt. Besides the broad distribution range, large sampling, which covered most of the natural distribution records, was also a fundamental factor. Another reason may be the long evolutionary history of *C. serrulata*, which was dated back to 8.84 mya, earlier than *C. dielsiana* (6.28 mya; Zhu et al., 2019), *Panzerina lanata* (Lamiaceae) (1.61 mya; Zhao, Zhang, Pan, & Zhang, 2019), and *Quercus glauca* (Fagaceae) (0.92 mya; Xu et al., 2015). This long existence allowed a more significant accumulation of variants, especially strengthened by a wide and strong adaptation.

### 4.2 Genetic differentiation and structure

Genetic differentiation is related to gene flow, natural selection, and mutation. Gene flow can promote genetic exchanges between different populations and thus decrease population differentiation (Slatkin, 1987). Plant gene flow mainly consists of pollen flow and seed flow (Dick, Hardy, Jones, & Rémy 2008). Therefore, the dispersal of seeds and pollen plays an important role in gene flow. Generally, cross‐pollinated plants and plants whose seed dispersal assisted by animals have higher gene flows (Loveless & Hamrick, 1984), while geographic obstacles such as mountains, seas, and rivers will impede gene flow (Slatkin, 1987). Natural selection usually happens in a specific environment which is distinct, and which puts high pressure on a species to evolve to adapt to the special environment. Thus, natural selection will strengthen specialization and increase genetic differentiation (Ehrlich & Raven, 1969; Endler, 1997). Mutation is the ultimate origin of all genetic variation (Barton, 2010); therefore, it provides the basic source of genetic differentiation.

In this study, AMOVA revealed a high genetic differentiation level among *C. serrulata* populations (cpDNA: *F*_st_ = 0.72461; ITS: *F*_st_ = 0.88730), and the most dominant source of variations came from variation among regions (cpDNA: 67.78%; ITS: 87.60%), signifying the existence of a phylogeographic structure (*N*_ST_ = 0.388 > *G*_ST_ =0.328, *p* < .05). A similar high diversity level was also detected in *C. dielsiana* (ITS: *F*_ST_ = 0.7827; Zhu et al., 2019), but contrarily low levels were unraveled in *C. pseudocerasus* containing both wild populations and landraces (cpDNA: *F*_ST_ = 0.24922, ITS: *F*_ST_ = 0.31571; Chen et al., 2015), *C. tomentosa* (SSR: *F*_ST_ = 0.181; He et al., 2015), *C. lannesiana* var. *speciosa* (cpDNA: *F*_ST_ = 0.1655; Kato, Iwata, Tsumura, & Mukai, 2011), *C. mahaleb* (RSPD: *F*_ST_ = 0.1646; Jordano & Godoy, 2000), *C. campanulata* (SSR: *F*_ST_ = 0.1477; Lv, 2006; Su, 2007), and *C. jamasakura* (SSR: *F*_ST_ = 0.043; Tsuda et al., 2009). This might be because both *C. serrulata* and *C. dielsiana* are much more widely distributed than most of the species of *Cerasus*. The various habitat environments have pressed different natural selections on populations and have gradually caused differentiation among them. In addition, the long distance and some geographic obstacles, such as sea and mountains, had limited gene flows between them and made them unable to homogenize the diverse genetic materials, and thus, a high genetic differentiation level was generated.

A total of six geographic groups (or regions) were identified from the 18 populations of *C. serrulata*. The grouping of Region I in the southwest (population MP, BTM, and LiS in the Qinling Mountains and the Taihang Mountains) and Region VI in the junction of China and the Korean Peninsula (population FHS and K‐NS) was the same in both DNA fragment analyses. Grouping of the populations slightly differed in the treatment of the two populations by cpDNA sequences and ITS (nuclear) sequences: HeS and TTZ (Tables 1, 2, Figures 1, 2). The two populations are located, respectively, in the two junctions of Region III and Region II, and Region III and Region V. This indicates some genetic associations between these three regions. Clustering of haplotypes of cpDNA sequences according to the distribution pattern, NJ tree, and network was similar to the grouping of populations, but differed in the clustering of haplotypes of the three regions. Generally, haplotypes of these three regions were clustered into two groups because of the close association and constant distribution. More simply, clustering of ribotypes of ITS sequences grouped ribotypes of the three regions together into one single group, also including Region IV in the Wuyi Mountains with one population HGS. The genetic relationship of the three regions is further confirmed by analyses of genetic differentiation and gene flow (Tables 4, 5). High and affluent gene flow (significantly larger than 1) was detected among Region II and Region III, and Region III and Region V. It indicated that Region III reacted as a transition between Regions II and V and connected these regions together. Taken together, Regions II, III, V, and IV should be incorporated into one lineage.

However, the lineage origin of Region VI concerning the junction of China and the Korean Peninsula (population FHS and K‐NS) remained unclear. Haplotypes of cpDNA within this region showed a closer relationship to Region V, and the two regions exchanged relatively sufficient gene flow (Figure 1, Table 4). This phenomenon was also observed in analyses using nuclear SSRs (Chen, 2016; Yi et al., 2018). But the ribotypes of nuclear ITS were clustered with Region I in the northwest region and shared little gene flow with Region V (Figure 2, Table 5). This confusion may be because of the less sampling of ITS sequences. Furthermore, actually, distributions of *C. serrulata* were also recorded in Hebei Province and Heilongjiang Province in China, as well as in Japan. But we failed to obtain samples in these regions. Thus, a lack of comprehensive sampling also caused this confusion.

Taken together, two lineages of the populations of *C. serrulata* were inferred. One is in the northwest region with group MP, BTM, and LiS. This lineage is confined to the Qinling Mountains and the Taihang Mountains. The other evolved from the southwest region (around the Wuling Mountains), eastward through the Jiangnan Hilly Region, and northward to the plain on the east coast of Asia. Populations around the junction of China and North Korea, as well as in the Korean Peninsula, remained unclear and needed more comprehensive evidence.

Region I in the southwest region, located in the Qinling Mountains and the Taihang Mountains, appeared to have a relatively isolated condition. This region was the first to diversify out from others, at about 8.84 mya (Figure 3). This was within the time of the Himalayan movement, which might have caused uplifting of the Qinling Mountains (Xue, Li, Li, & Liu, 2004) and might have isolated the region from others later on. The long isolation without sufficient gene flow (Tables 4, 5) gradually resulted in the differentiation of this region.

Region IV (population HGS, in the Wuyi Mountains) showed a very distinct differentiation pattern. The environment of this region is very special, as it is an alp, with low temperatures and high humidity. This specialized environment should have put high selection pressure on this population, and the high altitude reduced gene flow from other populations. Thus, the distinct differentiation in Region IV was generated.

### 4.3 Demographic dynamic and potential glacial refugia

The most recent common ancestor of *C. serrulata* appeared around 8.84 mya in the mid‐ to late Miocene, as calculated in the molecular dating analysis (Figure 3). Of the two lineages of *C. serrulata*, the crown time of the first lineage, in Region I in the northwest, was around 3.93 mya; the crown time of the other lineage, distributed from the southwest to northeast, is dated to around 7.27 mya. In other species, a similar time was also detected for the *C. dielsiana* ancestor (6.28 mya; Zhu et al., 2019), the *Polystichum glaciale* ancestor (6.89 mya; Dong, Xu, Rana, Li, & Sun, 2018), and the divergence of *Ixiolirion tataricum* and I. songaricum (~7 mya; Li, Song, Zhang, & Lv, 2019). It seems that the divergence event of many species was concentrated during such a period of time, which overlapped with Himalayan movement, the beginning of the development of the Arctic ice sheet, and when the global climate becoming cooler (Liu, Zheng, & Guo, 1998) and drier (Ma & Tian, 2015; Zhao, Lu, & Tang, 2018). These strong landform and climate changes might lead to such a concentration of species divergence. As such, the uplifting of the Qinling Mountains might have taken place accompanied by Himalayan movement (Xue et al., 2004) and might have gradually isolated the north groups from others, finally resulting in the divergence of the two geographic lineages.

No dominant expansion events were recognized either in the neutrality test or in the mismatch distribution analysis of different regions at the species level, indicating that *C. serrulata* might have reached neutrality in recent times. The genus *Cerasus* is reported to have formed its distribution center and pattern before the Last Glaciation Maximum (LGM, from 0.0265 mya to 0.019–0.02 mya) (Cao, 2006; Li, 2009; Wang, 2014). Thus, we would like to infer that *C. serrulata* also finished its distribution center and pattern before the LGM, experienced no significant colonization, and stayed relatively stable thereafter.

Glacial refugia, which protected species and enabled them to survive, are usually considered to be large mountains. Nevertheless, more and more attention is being given to the importance of microrefugia, such as smaller massifs or lowland sites (Suchan, Malicki, & Ronikier 2019; Zhu et al., 2019). It is believed that the broad land of the southeast of Asia was linked together by the Bohai Sea and the coast of Asia during glaciation, when sea levels declined (Guo et al., 2014). This area provided corridors for plants and animals, allowing them to travel between areas that are now broken up by the Bohai Sea. As such, the genetic link of populations in the Korean Peninsula and populations in the coast of east Asia can be explained. Valleys in the east–west mountains, such as the Qinling Mountains, could provide refugia and travel access for many species (Wulufu, 1964; Zhou, 1997). As such, the large mountains of the Qinling, the Wuling, and the Taibai Mountains in Korea, as well as some microrefugia in the Jiangnan Hilly Region, especially the Huangshan Mountains, Mount Lu, and Tianmu Mountain, were inferred to be glacial refugia for *C. serrulata*, also having regarded their higher genetic diversity. All the lineage colonizations and potential refugia are generally coincident with the result of phylogeographic analysis of *Cerasus* (Li, 2009), indicating that the widespread of *C. serrulata* can be a representative species of the genus *Cerasus* in the evolutionary process.

### 4.4 Taxonomic reconsideration

*Cerasus serrulata* is a complex with ample variations and transition morphological characters which have brought great taxonomic controversies. Here, we sampled two controversial groups of this complex to reveal the relationship. *C. laoshanensis*, published in 2017, was described to resemble *C. serrulata* (Zang, 2017). In this study, population LaoS was sampled to represent *C. laoshanensis*. But it showed no specificity from *C. serrulata* individuals in any relationship analysis (Figures 1, 2 and Tables 1, 2). Thus, it was endorsed to merge *C. laoshanensis* into *C. serrulata*. As for *C. huangangensis*, a record in 2007 (Yi, 2007), represented by population HGS in this study, showed distinctiveness as it exclusively possessed only one kind of haplotype or ribotype of both cpDNA and ITS sequences (Figures 1, 2 and Tables 1, 2). But it was still well mixed within the big clade in both phylogenetic analyses of NJ trees (Figures 1b, 2b) and ML tree (Figure 3) of *C. serrulata*. Therefore, better treatment of it would be a variety: *C. serrulata* var. *huangangensis*.

### 4.5 Implications for conservation and utilization

Flowering cherry possesses good ornamental value and is popular around the world. Nowadays, cultivars of flowering cherries are mainly developed from Japan. According to our accumulated observation and study on the cherry resources of China and Japan, we believe many of the good cultivars have some origin from *C. serrulata*. There are abundant *C. serrulata* resources in China. Making good use of this efficient resource will contribute significantly to new cultivar breeding. However, conservation exists side by side (and plays a part together) with utilization of resources. Despite detecting a high genetic diversity at the species level, diversity levels of many populations and some regions are not remarkable and some even show no diversity (Tables 1, 2). In addition, the actual wild situation for *C. serrulat*a is not good. *C. serrulata* is generally not dominant in communities and grows at marginal areas in forests with a relatively weak competitiveness. With the development of human activities, many of the resources have been damaged and lost. Thus, conservation and collection of these resources are of great significance. Both lineages should be collected. The first lineage, in the Qinling Mountains and the Taihang Mountains (Region I), is relatively isolated and ancestral, and the genetic diversity is low, which needs protection. Populations in the southwest region, especially population CPL in the Wuling Mountains (Region II), are most likely to be a current genetic diversity center as the region shows the highest genetic diversity. Furthermore, the origin is relatively ancestral. Thus, this region deserves attention regarding source collection and utilization for cultivar development. Populations in the Jiangnan Hilly Region (Region III) and the Korean Peninsula and the junction of China and North Korea (Region VI) also possess high genetic diversity, which is also worth attention. Furthermore, Region VI is located northernmost and may be valuable in developing cultivars with high cold resistance. Populations on the plain of the coast of east Asia (Region V) show relatively low diversity, which may be because of strong human interference. This resource needs practical protection measures and collection to avoid further resource loss. Habitats of HGS population in the Wuyi Mountains (Region IV) are the most special, as they are sited in alps with low temperature and high humidity. The unique environment should have pressed strong selection on this population, and the high altitude has prevented gene flow from other populations; thus, the analysis showed no genetic diversity of this population. Therefore, protection combining in situ conservation and ex situ conservation is suggested for this resource. Phenotypic characteristics of this population are also unique as the leaves are red and the plant is short and shaped like a shrub. Resources in this region have potential value for developing new cultivars with wet resistance, lower shape, and colored leaves.

## 5 CONCLUSION

We found high genetic diversity and the existence of phylogeographic structure in *Cerasus serrulata*. No dominant expansion events were detected. Finally, two geographic lineages were inferred. One was confined to the Qinling Mountain and the Taihang Mountains. The other was from the Wuling Mountains to the Jiangnan Hilly Regions, and then went northeast to the east coast of Asia. Taxonomic treatments of several related controversial species were reconsidered. *C. laoshanensis* was suggested to be merged into *C. serrulata*. And a new variety, *C. serrulata* var. *huangangensis*, was preferred.

## CONFLICT OF INTEREST

None declared.

## AUTHOR CONTRIBUTION

**Xian‐Gui Yi:** Conceptualization (equal); Data curation (equal); Formal analysis (equal); Funding acquisition (equal); Investigation (equal); Methodology (equal); Project administration (lead); Resources (equal); Validation (equal); Visualization (equal); Writing‐original draft (equal); Writing‐review & editing (equal). **Jie Chen:** Formal analysis (equal); Investigation (equal); Methodology (equal); Validation (equal); Visualization (equal); Writing‐original draft (equal); Writing‐review & editing (equal). **Hong Zhu:** Data curation (equal); Investigation (equal); Methodology (equal). **Yong‐Fu Li:** Formal analysis (equal); Investigation (equal). **Xue‐Xia Li:** Formal analysis (equal); Investigation (equal). **Meng Li:** Formal analysis (equal); Investigation (supporting). **Yi‐Fan Duan:** Investigation (supporting); Methodology (supporting). **Lin Chen:** Investigation (equal); Methodology (equal). **Xian‐Rong Wang:** Conceptualization (equal); Funding acquisition (lead); Methodology (lead); Project administration (equal); Supervision (lead).

## Data Availability

DNA sequences: GenBank accessions MT220011–MT221165.
